# The Multiple Actions of Amygdalin on Cellular Processes with an Emphasis on Female Reproduction

**DOI:** 10.3390/ph14090881

**Published:** 2021-08-30

**Authors:** Adriana Kolesarova, Simona Baldovska, Shubhadeep Roychoudhury

**Affiliations:** 1 Department of Animal Physiology, Faculty of Biotechnology and Food Sciences, Slovak University of Agriculture in Nitra, 94976 Nitra, Slovakia; 2 AgroBioTech Research Centre, Slovak University of Agriculture in Nitra, 94976 Nitra, Slovakia; simona.baldovska@uniag.sk; 3 Department of Life Science and Bioinformatics, Assam University, Silchar 788011, India; shubhadeep1@gmail.com

**Keywords:** amygdalin, reproductive biology, medicine, ovary, hormone, proliferation, apoptosis, toxicity

## Abstract

The present review summarizes the current knowledge on the provenance and properties, metabolism and toxicity, mechanism of action, physiological, and therapeutic roles of amygdalin—a molecule present in the seeds of apricot and other plants—with an emphasis on the action of amygdalin on reproductive processes, particularly in the female. Amygdalin influences physiological processes including female reproduction at various regulatory levels via extra- and intracellular signaling pathways regulating secretory activity, cell viability, steroidogenesis, proliferation, and apoptosis. On the other hand, while being metabolized in the body, amygdalin releases significant amounts of cyanide, which may lead to acute health hazard in those individuals who may be at risk. Despite some contradictions in the available data about benefits and toxic effects of amygdalin, its potential applicability at low doses may present a promising tool for regulation of various reproductive and other physiological processes including disease management primarily in cancer phytotherapy, animal production, medicine, and biotechnology. However, further research involving carefully designed dose–response studies is required to overcome the possible side effects of amygdalin and assure its safety as a therapeutic agent.

## 1. Introduction

The ovaries play a fundamental role in reproduction as well as the production of hormones and the egg cells, or oocytes for fertilization. Problems within the complex feedback loops in the ovaries can result in infertility, pain, or hormonal imbalance. Multiple pathological processes can occur in the ovaries, including ovarian cancer, ovarian torsion, ectopic pregnancy, ovarian abscess, hormonal imbalance, or cysts [1]. Understanding the physiological and natural regulators of reproduction is important not only for the characterization, prediction, and control of reproductive processes but also for the prevention and treatment of reproductive disorders.

Apricot (*Prunus armeniaca* L.) is an important commercial crop and consumed worldwide. Apricots with a number of flavonoids and carotenoids are believed to possess antioxidant properties [2,3,4]. Apricot seeds also make an important contribution to the diet in many countries [5]. Amygdalin, a biomolecule present in the seeds of apricot and other fruits, gained wide popularity owing to its purported anti-cancer activity [6]. Around 5000 years ago, the Egyptian papyri mentioned the beneficial use of the bitter almonds derivatives in treating skin tumors. Thereafter, those derivatives came to be known as amygdalin, vitamin B17 or laetrile, and bitter almonds are considered one of the richest sources [7,8,9,10]. Ancient Romans and Greeks connected some therapeutic properties to those derivatives, too. Interestingly, some isolated populations and tribes all over the world—such as the Abkhazians, the Hopi and Navajo Indians, the Hunzas, the Eskimos, and the Karakorum did not have the incidence of cancer cases. It turned out that they had common a diet rich in amygdalin [7]. Consequently, many researchers and scientists all over the globe carried out various studies and clinical trials to prove the anticancer activity of amygdalin. Despite the beneficial effect of cyanide against cancer, it may cause several harmful side effects and lead to toxicity [6,11], especially from amygdalin tablets ingestion [12].

The present review attempted to summarize the recent information concerning the provenance and properties, metabolism and toxicity, mechanism of action, physiological and therapeutic roles of amygdalin with an emphasis on its action on female reproductive processes at various regulatory levels.

## 2. Provenance and Properties

Amygdalin is abundantly present in the kernels of various species of *Rosaceae* family such as in the bitter seeds of apricots, apples, almonds, peaches, cherries, plums, grains, millets, sprouts, and nuts [9,13,14,15,16,17,18]. Although amygdalin was first discovered in 1803, it was isolated from bitter almonds only in the year 2003. Seeds contain amygdalin depending on the variety: apricot kernels and bitter almond kernels have a 3–4% content of amygdalin by weight and it may even rise up to 8% in apricot seeds [19]. Amygdalin content is particularly high (more than 5%) in bitter apricot cultivars [20,21,22,23,24]. Amygdalin contents of seeds from 15 varieties of apples ranged from 1 mg/g to 4 mg/g, however, the content is low in commercially available apple juice, ranging from 0.01 to 0.04 mg/mL for pressed apple juice and 0.001–0.007 mg/mL for long-life apple juice [15].

**Figure 1:**
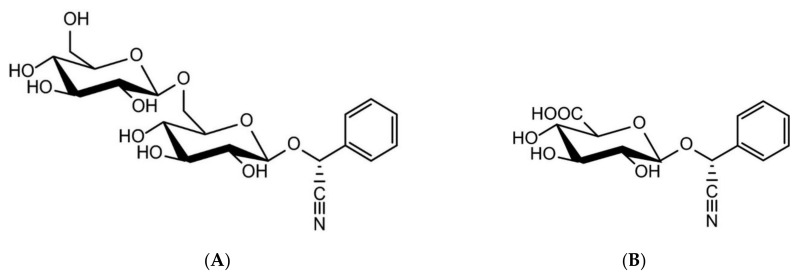
Chemical structures of amygdalin (**A**) and laetrile (**B**) [30].

Amygdalin is an aromatic cyanogenic compound belonging to the sub-class of carbohydrates and carbohydrate conjugates. The structure of amygdalin comprises one unit of benzaldehyde, one unit of hydrocyanic acid, and two units of glucose [25,26]. The chemical formula of amygdalin is C_20_H_27_NO_11_. Having a molecular weight of 457.432 g/mol, amygdalin is colorless with a melting point of 213 °C, insoluble in non-polar solvents like chloroform, and is highly soluble in ethanol and moderately soluble in water. It has D-mandelonitrile-beta-D-gentiobioside structure. However, the active form of amygdalin is R-amygdalin, a right-handed structure that is its natural form (Figure 1) [6,18]. Although known as vitamin B-17 or laetrile, from a biomedical point of view they are not the same product as amygdalin. In fact, amygdalin is a cyanogenic glucoside, and its purified form is called laetrile which refers to the terms levorotatory and mandelonitrile. Laetrile is a semi-synthetic cyanogenic glucuronide, and therefore it is structurally different from amygdalin [6,27,28,29]. Vitamin B17 is also a misnomer as amygdalin is not considered as a vitamin in strict sense [30].

## 3. Metabolism and Toxicity

Amygdalin is the major cyanogenic glycoside present in apricot kernels and is degraded to cyanide by chewing or grinding. Degradation of 1 g of amygdalin liberates 59 mg hydrogen cyanide (HCN) which is present in its dissociated form as cyanide. Moreover, cyanide is of high acute toxicity in humans [30].

Orally administered amygdalin is degraded into prunasin as the major metabolite by digestive enzymes after passing through the salivary and gastrointestinal phases. Prunasin is degraded into the mandelonitrile by β-glucosidase and then hydroxylated across the small intestinal wall—producing hydroxymandelonitrile (149 Da) under the glucosidase action, such as amygdalase and prunase—and ultimately decomposed into benzaldehyde and hydrogen cyanide [31,32,33].

Amygdalin itself is non-toxic, but its product HCN decomposed by some enzymes is a poisonous substance [34]. Recent studies have shown that HCN is released in normal cells, and therefore it may not be safe for the human body [35]. Serious side effects are caused by cyanide compounds liberated after amygdalin degradation [11,28]. Cyanide reversibly binds to ferric ion in cytochrome oxidase a3 within the mitochondria, effectively halting cellular respiration by blocking the reduction of oxygen to water [36]. The toxicity of cyanide is largely attributed to the cessation of aerobic cell metabolism and cellular hypoxia, which causes central nervous system and cardiovascular dysfunctions [37].

Cyanogenic glycoside as natural plant toxicants including amygdalin might present a potential risk for animal health [15,22,24]. Amygdalin-induced reactive oxygen species (ROS) production, and the subsequent benzaldehyde overproduction, may trigger protein oxidation before the lipid peroxidation process. Although low and medium doses (50 and 100 mg/kg) of orally administered amygdalin induce no toxicity in mice, amygdalin at a high dose (200 mg/kg) is capable of inducing toxicity and exerts negative effects on the oxidative balance of hepatic tissues with an obvious effect on the histopathology in mice [38].

Despite this fact, animal data do not provide any solid basis to support acute human health hazard assessment. Moreover, Bolarinwa et al. (2015) reported that amygdalin contents of commercially available apple juices are unlikely to present health problems to consumers [15]. The European Food Safety Authority’s Panel on Contaminants in the Food chain (CONTAM Panel, 2016) concluded that the lethal dose is 0.5–3.5 mg/kg of body weight (b.w.). An acute reference dose (ARfD) of 20 μg/kg b.w. is derived from exposure of 0.105 mg/kg b.w. associated with a non-toxic blood cyanide level of 20 μM, applying an uncertainty factor of 1.5 to account for toxicokinetic and of 3.16 to account for toxicodynamic inter-individual differences [30]. The highest dose of amygdalin that does not cause any unacceptable side effects in mice, rabbits, and dogs is 3 g/kg injected intravenously and intramuscularly, and 0.075 g/kg when given orally. Also, the maximum tolerated dose of human intravenous injection of amygdalin is around 0.07 g/kg.

Interestingly, in the mice treated by inhibiting the intestinal bacteria, the oral administration of 300 mg/kg b.w. did not lead to death. On the other hand, the mortality increased by 60% using the same dose in untreated mice. Moreover, systemic toxicity has been reported in humans following oral administration of amygdalin at a dose of 4 g per day through a period of half a month, or a month of intravenous injection. The response of the digestive system toxicity is more frequent and accompanied by changes of atrial premature beats. Nevertheless, after the cessation of amygdalin intake or when the daily oral dose is reduced to 0.6–1 g, the toxicity disappeared [16].

## 4. Mechanism of Action

Previous studies suggested that amygdalin can impact numerous signaling pathways, which play pivotal roles in a variety of physiological and/or pathological processes including aberrant regulation as has been identified in various human diseases. Amygdalin inhibits the adhesion of breast cancer cells, lung cancer cells, and bladder cancer cells by decreasing the expression of integrins, reducing catenin levels, and inhibiting of the Akt-mTOR (mammalian target of rapamycin) signaling pathway, which may consequently lead to the inhibition of metastases of cancer cells [35]. Moreover, β-glucosidase can accelerate the hydrolysis of amygdalin into hydrogen cyanide, which can effectively kill tumor cells by inhibiting cytochrome C oxidase in mitochondria, resulting in a significant increase in the cell mortality rate [39].

The anti-cancer activity of amygdalin in renal cancer cells was described by increasing p19 protein expression, which results in inhibition of cell transfer from G1-phase to S-phase, and thus inhibits cell proliferation [35]. In vitro experiments have shown induction of apoptosis by amygdalin as a result of increased expression of Bcl-2-associated X protein (Bax) and caspase-3 proteins and reduced expression of anti-apoptotic B-cell lymphoma 2 (Bcl-2) protein [18,35]. Amygdalin exerts cytotoxic activities and induces apoptosis in estrogen receptors (ER)-positive MCF7 cells, and MDA-MB-231 and Hs578T triple-negative breast cancer (TNBC) cells. This natural compound downregulates Bcl-2, upregulates Bax, activates caspase-3, and cleaved poly ADP-ribose polymerase (PARP). It also activates a pro-apoptotic signaling molecule p38 mitogen-activated protein kinases (p38 MAPK) in Hs578T cells [40].

In addition, amygdalin alters cell cycle progression in bladder cancer cells by down-modulating cyclin-dependent kinase 2 (CDK2) and cyclin A. Amygdalin-induced suppression of CDK2, a key regulator of G1-S transition and modulation of G2 progression, induces cycle arrest in G0/G1 phase, thus inhibits cell proliferation and growth [41]. Amygdalin also shows dose-dependent effects on inhibition of hepatocellular carcinoma human cells (HepG2) proliferation and the ability to accelerate skeletal muscle cells (C2C12) growth by strong expression of mRNA follistatin, which is a regulator of muscle growth [42].

Amygdalin can compromise the oxidative balance. It decreases ROS production, levels of protein carbonyls and malondialdehyde (MDA) in the testicular tissue as a result of its antioxidant activity [43]. On the other hand, the mechanism of action against breast cancer cells is based on amygdalin-induced oxidative stress. Amygdalin is able to reduce the growth of MCF-7 and T47D human breast cancer cells in a concentration and time-dependent manner and causes the induction of the generation of MDA and oxidization of glutathione levels, as well as a decrease of total glutathione and glutathione reductase activity [44]. The modulatory properties of this substance are provided by decreasing the expression of cellular integrins β1, β4, integrin-linked kinase (ILK), and focal adhesion kinase (FAK) pathways, as well as β-catenin in lung cancer cells [45]. Other studies have indicated that amygdalin inhibits nuclear factor kβ (NF-kβ) and NLRP3 signaling pathways, and consequently exerts an anti-inflammatory effect due to a reduction in the expression of pro-inflammatory cytokines such as pro-interleukin (IL)-1β. Moreover, amygdalin inhibits proliferation and fibrosis of hepatic stellate cells (HSC-T6) by suppression of transforming growth factor β (TGF-β) expression, a key mediator of liver fibrosis. Also, the connective tissue growth factor (CTGF), which is regulated by TGF-β, is indirectly affected by amygdalin, therefore the anti-fibrotic effect of amygdalin may proceed via the suppression of the TGF-β/CTGF signaling pathway [46].

**Figure 2:**
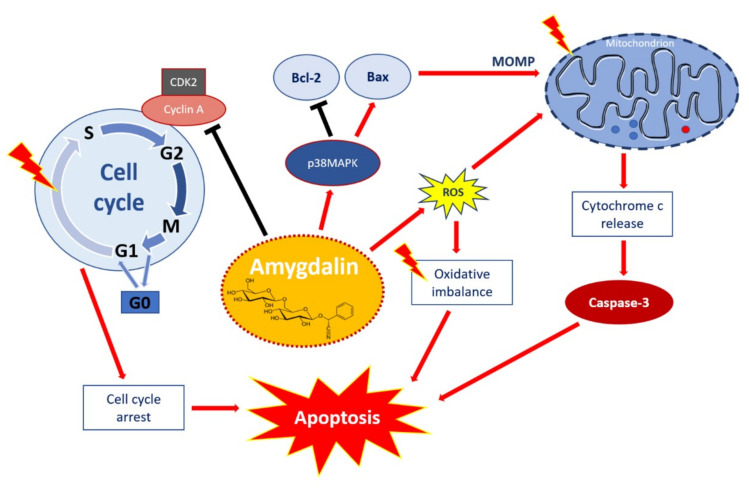
Apoptotic effect of amygdalin via multiple cellular signaling pathways. Amygdalin activates p38MAPK, which affects death stimuli: activates Bax apoptotic proteins, and inhibits Bcl-2 anti-apoptotic proteins. Apoptosis-related proteins induce mitochondrial outer membrane permeabilization (MOMP), a decisive event in the process of cytochrome c release. Activation of the release of cytochrome c as a mitochondrial response to proapoptotic stimuli via the mitochondrial or intrinsic apoptotic pathway ultimately leads to activation of caspases including caspase-3, which induces apoptosis. Amygdalin induces ROS overproduction, which compromises oxidative balance and eventually leads to apoptosis. Amygdalin down-regulates cyclin-dependent kinase 2 (CDK2) and cyclin A, which induces cell cycle arrest in G0/G1 phase. Amygdalin also inhibits cell transfer from G1 to S phase resulting in the inhibition of cell proliferation and growth. ‘Activation’ shown by red arrows and ‘inhibition’ showed by black arrows.

Taken together, the available data demonstrate that amygdalin may affect a wide array of cellular processes. It may be postulated that the mechanism of amygdalin against cancer cells is modulated mainly through the induction of oxidative stress and via multiple intracellular signaling pathways involved in the cell growth inhibition, reducing the proliferation and induction of apoptosis. The multiple signaling pathways involved in the apoptotic effect of amygdalin and associated with many cellular processes such as viability, cell growth, cell cycle control, as well as cell death are visualized in Figure 2.

## 5. Physiological and Therapeutic Roles

Natural substances and alternative medications such as amygdalin gained huge popularity in treating various diseases due to their wide availability and relatively low cost [6]. Numerous previous studies have documented that amygdalin has antibacterial, anti-inflammatory, and immunoregulatory activities, as well as improves the immune function of organism, and affects oxidative balance [25,38,47,48,49].

Amygdalin has been promoted as a natural anti-cancer agent. However, it is still unclear how this compound affects non-cancer cells. Amygdalin can decrease cell viability of primary human osteoblast in vitro and negatively affect osteoblasts morphology including mineralization of extracellular matrix and bone resorption. It also modulates the expression of important genes in human osteoblasts associated with osteoblast-specific pathways, oxidative stress, and cell death [50]. Subacute intramuscular exposure to amygdalin at doses of 0.6 and 3 mg/kg b.w. during of 28-days period affects compact bone remodeling including vascularization and biomechanical properties and causes evident changes in compact bone microstructure [51]. Moreover, long-term consumption of bitter apricot seeds 60 and 300 mg/kg b.w. administered daily during a 10-month period might affect the liver microscopic structure in rabbits. However, the toxic effect could not be accurately corroborated, as in many cases changes were dose-dependent and not recorded at the highest dose 420 mg/kg b.w. used in the study [24].

In contrast, in vivo studies were designed to reveal whether pure amygdalin or apricot seeds induce changes in the overall health status of rabbits as a biological model. Short-term application (2 weeks) of pure amygdalin intramuscularly injected at doses 0.6 and 3.0 mg/kg b.w. or oral consumption of crushed bitter apricot seeds (*Prunus armeniaca* L.) at doses 60 and 300 mg/kg b.w. did not represent a risk for animal health from the perspective of biochemical and hematological parameters, as well as antioxidant enzyme activity, and endocrine profile [23,52]. Additionally, it has been revealed the beneficial properties of bitter apricot seeds with the content of amygdalin on the risk of cardiovascular diseases. After 12 weeks of daily consumption of bitter apricot seeds (60 mg/kg b.w.), changes in the lipid profile of the volunteers were reported. Therefore, regular intake of bitter apricot seeds may be considered potentially useful for the prevention of cardiovascular diseases [20]. Furthermore, the protective effect of amygdalin in the treatment and recurrence of endometriosis has been reported [53]. Additionally, semen *Armeniacae amarum* (a Chinese traditional medicine with its amygdalin content) is known to exert antitussive and anti-asthmatic effects [32]. It was confirmed that amygdalin (at the concentration of 200 µmol/L) stimulates the proliferation of hyperoxia-exposed type II alveolar epithelial cells (AECIIs) isolated from premature rat lungs in vitro, thus amygdalin may have a protective effect in hyperoxia-induced premature lung injury [33]. Furthermore, the effect of amygdalin in the spinal cord is associated with suppression of pro-inflammatory cytokine release. Amygdalin inhibits c-Fos, tumor necrosis factor α (TNF-α), and IL-1β expression as results of its anti-inflammatory and analgesic properties [17]. The antioxidant activity of amygdalin is associated with a decrease in ROS production and protein and lipid oxidation. On the contrary, long-term amygdalin administration (28 days) at high doses (3.0 mg/kg b.w.) can increase protein oxidation and lipid peroxidation as a result of its ability to compromise oxidative balance in the testicular tissue, as well as can increase the ROS production, which leads to cellular oxidative stress [43].

Amygdalin possesses therapeutic effect against atherosclerosis [9], in the management of autoimmune hepatitis [54], and anti-tumor effects against various cell lines [13,16,25,35,41,55,56,57]. This phytocompound can affect the cell cycle of cancer cells, reduce cell cycle activators, especially cyclin B, cdk1, E-cadherin, and N-cadherin and activate multiple cellular pathways, inhibit the Akt-mTOR signaling pathway thereby inhibiting the proliferation of cancer cells [45,56,58]. Moreover, the mechanism of action of its anti-tumor activity relates to the ability of amygdalin to induce cell apoptosis through regulation apoptotic proteins, increasing the level of proapoptotic Bax proteins, inducing activity of caspase-3, and decreasing the level of anti-apoptotic protein Bcl-2 [18,35].

Although amygdalin has a clear pharmacological activity, there is still little in-depth research on the pharmacological mechanism of this compound [16]. Amygdalin as a therapeutic agent does not have widespread use across the globe owing to insufficient clinical verification of its therapeutic efficacy and adverse side effects. It was suggested in targeted enzyme/pro-drug strategies as a means to improve the tumor selectivity of therapeutics with decreased side effects [59].

Although the characteristic action of amygdalin remains controversial, systematic investigation of the mechanism of its pharmacological activity may aid the development of anti-tumor drugs. For better visualization of the data of amygdalin’s physiological and therapeutic actions, the in vivo and the in vitro effects of amygdalin are summarized in Table 1.

## 6. Effects on the Female Reproduction

There is a significant amount of evidence for the role of amygdalin on male reproductive functions in animals [21,22,38,43,60], as well as female reproductive system in vivo and in vitro [23,53,61,62,63,64]. Amygdalin application positively affects ovarian cell viability and stimulates testosterone release by porcine ovarian granulosa cells; therefore, amygdalin can exert a potential effect on cellular growth and the process of ovarian steroidogenesis in vitro [62]. In addition, amygdalin has an evident effect in the treatment of endometriosis, an aggressive disorder associated with infertility, pelvic pain, and intra-abdominal adhesions in women of reproductive age [53].

Previous studies by our group have reported the effects of amygdalin on endocrine regulation of ovarian functions and secretion activity of porcine ovarian granulosa cells focused particularly on the process of ovarian steroidogenesis. The modulatory effect of amygdalin on steroid hormone secretion by ovarian cells was noted [23,62,64]. Furthermore, amygdalin administration caused a dose-dependent stimulation of 17β-estradiol but not of progesterone release by porcine ovarian granulosa cells [61]. The results have suggested the possible involvement of this natural substance into the processes of steroidogenesis—and amygdalin, in combination with the mycotoxin deoxynivalenol (DON) can also exert the possible positive effects on the steroid hormone secretion (progesterone and 17β-estradiol) by porcine ovarian granulosa cells. Interestingly, solely the presence of pure amygdalin causes the stimulation of 17β-estradiol secretion, and amygdalin appears to be a potential endocrine modulator in porcine ovaries. On the other hand, the results do not confirm the expected protective effect of pure amygdalin on mycotoxin-induced reprotoxicity [61,63].

Short-term intake of amygdalin at recommended doses of 0.6 and 3.0 mg/kg b.w. does not present risks for animal health from the perspective of biochemical parameters including endocrine profile. No obvious beneficial or negative effects of amygdalin on the physiological functions of female rabbits were demonstrated and no clinically noticeable changes in the average body weight of experimental animals were observed [52]. Another in vivo study was aimed at demonstrating whether amygdalin is able to cause changes in the endocrine profile and thus alter the key reproductive and physiological functions, using rabbit as a biological model. Plasma levels of steroid (progesterone, 17β-estradiol, testosterone), thyroid (triiodothyronine, thyroxine, thyroid-stimulating hormone), as well as anterior pituitary (prolactin, luteinizing hormone) hormones were assessed with no significant results. Intramuscular amygdalin application does not affect the plasma levels of selected endocrine regulators. Similarly, the oral form of amygdalin did not induce significant changes in the plasma levels of examined hormones either [23].

Taken together, the above-mentioned findings indicate the influence of amygdalin on physiological processes including female reproduction at various regulatory levels via extra- and intracellular signaling pathways regulating secretory activity and steroidogenesis, as well as compromise the delicate oxidative balance in various cells and reproductive tissues (Table 2).

## 7. Application in Reproductive Biology and Medicine

At this time, little is known regarding the therapeutic doses of amygdalin. Extensive literature search did not reveal any study on the effects of amygdalin on either the female gametes (oocytes) or the male gametes (sperm). Treatment time and amygdalin application type may also vary between humans and animal models. Moreover, there have been reports of cyanide toxicity due to amygdalin uses and large doses of amygdaline may cause systemic toxicity, which limits its clinical application [16,38,65].

Specific activation of amygdalin by β-glucosidase in tumor tissue may be an effective method for decreasing the general toxicity and increasing the killing effect [59]. The enzyme or its encoding gene is first delivered to the tumor site using a targeting carrier. After clearance of the enzyme from circulation, the prodrug is administered and then converted to an active anti-cancer drug, thus achieving enhanced anti-cancer efficacy and decreased systemic toxicity [66,67]. The toxic effect of cyanide is associated with the cytochrome oxidase terminal in the mitochondrial respiratory pathway, which hinders the ability of cells to use oxygen [68].

Amygdalin can act as a pro-oxidant, as well as can modulate the oxidative balance instead of ROS overproduction [69]. Unlike normal cells, in cancer cells, the high levels of ROS result in mitochondrial dysfunction and increased metabolism. This mechanism is related to the anti-tumor effects of amygdalin by triggering several ROS-induced cell death pathways of cancer cells [70]. Effects of amygdalin on oxidative stress parameters and oxidant and/or antioxidant properties of amygdalin and bitter or sweet apricot kernels have been described by previous authors [38,43,44,69].

Intramuscular amygdalin administration causes changes in the oxidative profile of rabbits and affects the testicular tissue in a dose-dependent manner. Amygdalin acts as an antioxidant at low doses (0.6 mg/kg b.w.) while high doses (3.0 mg/kg b.w.) compromise the delicate oxidative balance in male reproductive structures [43]. The highest efficacy of orally administered amygdalin was noted at a moderate dose of 100 mg/kg by enhancement of antioxidant enzyme activities, including upregulation of the expression of glutathione peroxidase and superoxide dismutase, and decreasing in lipid peroxidation levels in hepatic and testicular tissues thereby improving oxidative balance [38].

Intramuscular and oral application of amygdalin does not appear to have any negative impact on the plasma levels of certain endocrine regulators (progesterone, 17β-estradiol, testosterone), thyroid (triiodothyronine, thyroxin, thyroid-stimulating hormone), anterior pituitary hormones (prolactin, luteinizing hormone), or the average body weight of rabbits [23].

As mentioned earlier, the beneficial properties and health-promoting effects of amygdalin particularly on the treatment and recurrence of endometriosis have been reported [53]. Similarly, analogues of amygdalin with cyanide group removed may play a valuable role in improving the immune function of the organism [49]. Amygdalin significantly increases polyhydroxyalkanoates which stimulates peripheral blood T-lymphocytes to secrete IL-2 and interferon γ (IFN-γ), and then reduce the secretion of TGF-β1 [16]. In addition, this phytocompound is involved in the regulation of female reproductive functions through endocrine regulators such as TGF-β signaling pathway [16] and ovarian steroid hormones [61,62,63,64]. The studies have revealed the potential impact of amygdalin on cellular growth as well as its mechanism of action in the process of ovarian steroidogenesis. Thus, amygdalin application can regulate ovarian cell viability and secretory activity [61,62,63,64].

**Figure 3:**
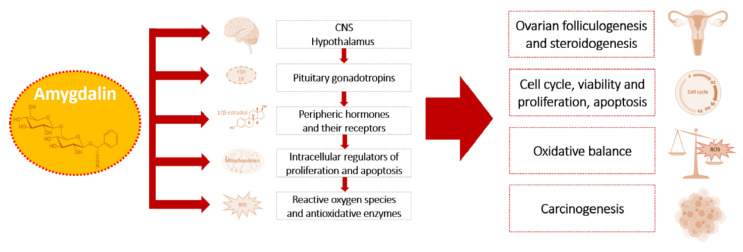
Targets and physiological actions of amygdalin on female reproductive processes and their pathological alterations.

Available data demonstrate that amygdalin possesses anti-tumor activity in a dose-dependent manner in various cells and affects the process of carcinogenesis, as well as female reproductive processes via the system of the hypothalamic–pituitary–ovarian axis, its hormones, growth factors and their receptors, and through the modulation of oxidative balance [13,16,25,41,53,57,61,62,63,64]. Mentioned targets and mechanisms of amygdalin action on the female reproductive system are summarized in Figure 3.

Understanding the toxicity and efficacy of this compound may advance the development as a therapeutic agent and determination of safe and effective oral doses of amygdalin. However, further research is necessary to validate the exact physiological effects of amygdalin on healthy cells vis-à-vis cancers and other diseases for potential clinical application.

## 8. Conclusions and Possible Directions of Further Studies

The review summarizes the recent information concerning the properties, metabolism, physiological role, toxicity, and therapeutic potential of amygdalin with an emphasis on its action on female reproductive processes at various regulatory levels. Despite some contradictions, the available data indicate towards its beneficial impact on the organism, as well as cytotoxic effects on cancer cells, the applicability of amygdalin at low doses as a promising therapeutical agent for regulation of various reproductive and non-reproductive processes and prevention and treatment of various disorders primarily in the anti-cancer therapy, reproductive medicine, and biotechnology.

Overall, amygdalin and its metabolites could be a tool to control female reproductive processes, including prevention or treatment of reproductive disorders. Knowledge about this biologically active compound is insufficient for adequate understanding and potential use against female reproductive disorders. Therefore, amygdalin represents promising bioactive phytocompound for basic and applied research in the future.

## Figures and Tables

**Table 1:** Physiological and therapeutic actions of amygdalin.

Dose of Amygdalin	Treatment Time	Experimental Model	Actions	Ref.
		In vivo studies		
1, 3, 10 mg/kg/day i.p.	4 weeks	mice	protective effect against atherosclerosis	[9]
5 mg/kg/week i.p.	3 weeks	mice	protective effect against autoimmune hepatitis	[54]
0.1, 0.5, 1 mg/kg i.m.	30 min	rats	analgesic effect	[17]
50, 100 mg/kg orally	2 weeks	mice	antioxidant effect	[38]
0.5, 1, 2 mg/kg i.p.	1 h	mice	anti-inflammatory effect	[47]
2, 4, 8 mg/kg i.p., twice (12 h interval)	48 h	mice	anti-inflammatory effect	[48]
320 mg/kg every 2 days, i.t.	17 days	C57BL/6 mice	anti-tumor effect	[55]
		In vitro studies		
200 µmol/L	24 h	premature rats AECII	protective effect against lung injury	[33]
200 μg/mL	48 h	rat hepatic stellate cells	anti-fibrotic effect	[46]
10^−8^ and 10^−6^ M	48 h	human keratinocyte cells	immunomodulatory effect	[49]
65 mmol/L	48 h	human breast cancer cells	anti-tumor effect	[44]
2.5 and 5 mg/mL	48 h	human lung carcinoma cells	anti-tumor effect	[45]
10 mg/mL	24 h	mouse prostate cancer cells, human prostate cancer cells	anti-tumor effect	[55]
10 mg/mL	24 h	renal carcinoma cells, bladder cancer cells, prostate cancer cells	anti-tumor effect	[41,56,57,58]

**Table 2:** Physiological and therapeutic actions of amygdalin on reproductive processes.

Dose of Amygdaline	Treatment Time	Experimental Model	Actions	Nature of Effect	Ref.
		In vivo studies			
100 mg/kg/day orally	2 weeks	male mice	antioxidant effect	positive	[38]
0.6 mg/kg b.w./day i.m.	28 days	male rabbits	antioxidant effect	positive	[43]
5 mg/kg/week i.p.	42 days	female rats	protective effect against endometriosis	positive	[53]
		In vitro studies			
0.5, 1, 2.5 and 5 mg/mL	5 h	rabbit spermatozoa	stimulatory effect	positive	[60]
1, 10, 100, 1000 and 10,000 µg/mL	24 h	porcine ovarian cells	stimulatory effect	positive	[61,62,63,64]

## Data Availability

No new data were created or analyzed in this study. Data sharing is not applicable to this article.
